# Evolutionary mismatch in emotional support during childbirth: Lessons from the COVID-19 pandemic

**DOI:** 10.1093/emph/eoae033

**Published:** 2024-12-16

**Authors:** Zaneta M Thayer, Anna Samsonov, Charlotte V Farewell, Theresa E Gildner

**Affiliations:** Department of Anthropology, Dartmouth College, Hanover, NH, USA; Department of Anthropology, Baylor University, Waco, TX, USA; Colorado School of Public Health, University of Colorado School of Medicine, Aurora, CO, USA; Department of Anthropology, Washington University in St. Louis, St. Louis, MO, USA

**Keywords:** physiologic birth, mismatch, social support, biomedicalization

## Abstract

**Background and Objectives:**

Selective pressures on human childbirth have led to the evolution of cooperative birth practices, with birth attendants playing a crucial role in providing emotional support during labor.

**Methodology:**

We leveraged COVID-19-related healthcare disruptions to investigate the impact of the evolutionary mismatch in the availability of emotional support persons on perceived birth stress among a US-based convenience sample (*N* = 1082).

**Results:**

Individuals who stated during pregnancy that they desired support from their partner or a doula but who did not receive this support had significantly higher perceived childbirth stress (*B* = 12.5, *P* < .0001; and *B* = 5.2, *P* = .02, respectively, measured on a scale of 0–100). The absence of any support persons (*B* = 6.7, *P* < .001), the number of emotional support persons present (*B* = −5.8 for each additional support person, *P* = .01), and the feeling that the healthcare provider was busy or distracted during labor (*B* = 15, *P* < .001) was significantly associated with childbirth stress. Virtual support did not attenuate these effects.

**Conclusions and Implications:**

Not being able to have desired emotional support during labor was associated with significantly higher childbirth stress, even after adjusting for clinical childbirth complications. These effect sizes were substantial, comparable to the elevated stress associated with cesarean section delivery and other childbirth complications. These findings underscore the importance of preventing an evolutionary mismatch in emotional needs during labor by ensuring access to continuous support, even during public health emergencies.

## BACKGROUND

Human childbirth has been strongly shaped by evolution. During parturition, the human fetus undergoes a uniquely complex rotational descent through the pelvis [1]. In response to this evolutionary selective pressure, humans have developed a reliance on cooperative and social birthing practices, a phenomenon termed “obligate midwifery” by Trevathan [2]. While some evidence of social birth exists in other primates (e.g. [3–5],), the near universality of this practice and the active role taken by birth attendants is believed to be a distinctive trait of our species. Trevathan postulated that social birth assistance is a product of natural selection, with individuals who sought aid having higher fitness. Consequently, birth support offered by kin and community members, particularly those with prior childbirth experiences, has become a consistent feature across diverse cultures [6].

Birth assistance carries profound implications during medical emergencies, such as cases where the umbilical cord is entangled around the newborn’s head [7], but this assistance extends beyond such critical situations. Other key types of support include informational support, advocacy, and emotional support [8]. Emotional support during labor, specifically support that fosters comfort and encouragement and makes the recipient feel loved and respected [9, 10], has been shown to trigger the release of oxytocin, a hormone that orchestrates uterine contractions during labor and initiates successful breastfeeding [11]. Physical touch from birth attendants also stimulates oxytocin release [12]. Mirroring its effects in non-parturient contexts, oxytocin fosters positive mood, reduces stress, and, crucially during labor, mitigates perceived pain [12]. Emotional support therefore exerts notable biological impacts on the labor and birthing process. For instance, a Cochrane review demonstrated that emotional support during labor corresponds to a reduced likelihood of negative childbirth sentiments, decreased use of intrapartum analgesia, shorter labor duration, and a lower chance of cesarean or instrumental vaginal births [13].

The biomedicalization of childbirth, which emphasizes a technocratic and medicalized approach to birth [14], has disrupted the availability of emotional support in labor. This biomedical model, which often deprioritizes emotional support and traditional birth practices, had particularly devastating effects during the COVID-19 pandemic. In an effort to control infection, many hospitals in the USA implemented strict policies that prohibited or severely limited the presence of preferred labor support persons, such as partners, mothers, or doulas [15, 16]. In some cases, individuals were forced to give birth without any emotional support persons due to hospital-imposed restrictions, support person infection, travel restrictions, or childcare needs [15]. Since these barriers to emotional support were somewhat randomly applied across birth locations and timeframes, the pandemic offers insight into how an evolutionary mismatch in the distinctly human reliance on emotional support during labor may impact perceived childbirth stress.

Here, we evaluate how giving birth alone, the number of emotional support persons, the absence of specific preferred persons, and the perceived availability of one’s medical provider are associated with perceived childbirth stress among individuals who gave birth during the COVID-19 pandemic in the USA,

## Methods

Data come from the COVID-19 and Reproductive Effects (CARE) study, which has been extensively described elsewhere [17]. In brief, this was an online convenience sample survey of pregnant people aged 18 years and older living in the USA. This study was approved by the Dartmouth Committee for the Protection of Human Subjects (STUDY00032045) and all participants provided informed consent. Participants were recruited through study announcements posted on social media platforms (Facebook, Twitter) and distributed via email to contacts working in maternity care and public health. The first survey, administered prenatally, was launched on 17 April 2020 using the Research Electronic Data Capture (REDCap) platform [18]. During the prenatal survey, participants provided their anticipated due date. Individuals who consented to be re-contacted were sent a follow-up survey to ask about their birth experience. The invitation for this postnatal survey was sent four weeks after their listed due date. Data for this analysis come from the prenatal and postnatal data collection waves. One thousand seven hundred and ninety-two participants from the pregnancy survey had complete data for the study variables and agreed to be contacted again. Of those, 1120 completed the postnatal survey (62.9%). Participants who completed the follow-up survey were more likely to have higher education but did not significantly differ from those who did not complete the follow-up survey in relation to age, self-identified race, prenatal depression, or previous birth. Complete data for all the study variables were available for 1082 participants.

### Dependent variable

#### Perceived birth stress

During the postpartum survey, participants were asked: “How stressful did you find the birth experience?” We used a visual analog scale (VAS) to allow participants to score from “Not at all stressful” to “Very stressful” on a scale of 0–100 (Supplementary Fig. 1). Previous research has found similar VAS scales are an easy-to-understand and implement measure, including when assessing childbirth-related stress and trauma [19].

### Independent variables

#### Give birth alone

During the postpartum survey, participants were asked, “Other than doctors, nurses, or midwives, who was in the delivery room with you when you delivered the baby? Check all that apply.” If individuals selected “no one” from the available options for this variable, then they were classified as having given birth alone.

#### Number of support persons

We summed the number of people that participants reported were in the room during delivery from the following options: partner, mother, father, sibling, friend, mother or father-in-law, a doula, or other. Given the small number of participants with four or five support persons (*N* = 6), we collapsed the available responses to 0, 1, 2, or 3 + for analysis.

#### Provider emotionally unavailable

To index the perceived emotional availability of providers, we asked whether during labor: “My care providers seemed busy/preoccupied/stressed, or had to limit their time in the room with me.” (Yes/No).

#### Mismatch in having specific support persons

During pregnancy, participants were asked: “If there were no restrictions, who would you ideally have in the room with you during delivery? Select all that apply.” They were able to select from the following options: Partner, parent, other family member, friend, doula, no one, or other. The three most common categories were partner (*N* = 1071), parent (*N* = 337), and doula (*N* = 204). We then used data on who individuals said was present during delivery to see if there was a “mismatch” in the desired presence for each of these three categories (mismatch partner, mismatch parent, mismatch doula).

### Covariates

#### Maternal age

Age (years) was analyzed as a continuous variable.

#### Race

Race was self-identified using US Office of Management and Budget categories (white, Black/African American, Asian, Hispanic, American Indian/Alaska Native [AI/AN], Other). Self-identified white ethnicity was used as the reference category for the race variable since this group was previously reported to have higher birth satisfaction than others during the pandemic [20].

#### Education

Education was analyzed as less than a college education, college-educated, or advanced degree.

#### Prenatal depression

The gold standard Edinburgh Depression Scale was used to assess maternal depression during the prenatal data collection wave. Individuals with an EPS score >=13 were categorized as having prenatal depression (yes/no) [21].

#### Nulliparous

Individuals were analyzed according to whether this was their first birth (yes/no).

#### Cesarean section delivery

Participants indicated whether they had undergone a cesarean section delivery (yes/no).

#### Labor and delivery complications

Participants indicated whether they experienced any complications during their labor and delivery (yes/no).

## Analysis

All analyses were performed using R version 4.2.3.

We first evaluated the characteristics of emotional support during delivery for our participants using descriptive statistics. We then ran six separate linear multivariate regression models to evaluate our hypothesis of whether emotional support predicted perceived childbirth stress. The first three variables were giving birth alone (dichotomous), number of emotional support persons (ordinal), and perceived emotional availability of providers (dichotomous). The next three models evaluated whether a mismatch in a desired partner, parent, or doula presence predicted perceived childbirth stress, with each of those variables analyzed dichotomously.

For all models, we evaluated multicollinearity (all variance inflation factors < 1.09), linearity, normality, and homoscedasticity to ensure all assumptions for linear regression were met. We set alpha at *P* < .05. Beta coefficients, 95% confidence intervals, *P*-values, and adjusted *R*^2^ values are reported for all models.

### Sensitivity analyses

We compared our longitudinally collected data on missing support persons with similar data collected cross-sectionally. During the postpartum survey we asked, “Was there anyone you wanted in the delivery room who was not there?” (Yes/No). If participants answered yes, we asked who was missing (options: partner, mother, father, sibling, a friend, mother or father-in-law, a doula, other). Finally, we asked “Was anyone able to attend the labor and delivery virtually (over a video chat or phone)?.” We used this to assess whether virtual labor support was associated with childbirth stress, or whether virtual support attenuated any of the associations with missing support persons and childbirth stress. This allowed us to evaluate whether in-person emotional support is particularly important for alleviating childbirth stress.

## Results

### Summary statistics and descriptive analysis

Sample characteristics are described in Table 1. The mean maternal age was 31.8 years (SD = 4.0; range = 18–47). Most participants had one support person at delivery (89.6%, *N* = 969); 1.9% (*N* = 21) had no support persons, 7.3% (*N* = 79) had two support persons, and 1.2% (*N* = 13) had three or more. Thirty-four percent of participants (*N* = 373) reported in the postpartum interview that there was someone they wanted at delivery who could not attend. Six percent (*N* = 72) of participants had at least one person attend the labor and delivery virtually, with those who were missing support persons being more likely to receive this form of support (12.6% [*N* = 47] of participants missing support persons vs 3.5% [*N* = 25] of those not missing a support person). 92.7% of participants who provided a reason for a missing support person (*N* = 346) said that at least one of the reasons that their missing support person(s) could not attend was due to hospital restrictions (See Table 2 for all listed reasons). Fourteen percent of participants reported that they perceived their provider as busy, worried, stressed, or limiting their time with them during labor.

**Table 1.** Summary statistics for sample. Continuous variables present mean (SD) and categorial variables *N* (%).

	All desired support persons (*N* = 709)	Missing desired support person (*N* = 373)	Overall (*N* = 1082)
**Perceived childbirth stress**	47.1 (28.3)	56.3 (28.8)	50.3 (28.8)
**Maternal age**	32.0 (3.77)	31.4 (4.47)	31.8 (4.03)
**Married/domestic partnership**	691 (97.5%)	350 (93.9%)	1041 (96.2%)
**Education**			
No college degree	95 (13.4%)	74 (19.8%)	169 (15.6%)
College degree	254 (35.8%)	130 (34.9%)	384 (35.5%)
Advanced degree	360 (50.8%)	169 (45.3%)	529 (48.9%)
**Self-identified race**			
White	637 (89.8%)	321 (86.1%)	958 (88.5%)
Hispanic	32 (4.5%)	23 (6.2%)	55 (5.1%)
Black/African American	7 (1.0%)	6 (1.6%)	13 (1.2%)
Asian	19 (2.7%)	12 (3.2%)	31 (2.9%)
American Indian/Alaska Native	2 (0.3%)	5 (1.3%)	7 (0.6%)
Other	12 (1.7%)	6 (1.6%)	18 (1.7%)
**Prenatal depression**	173 (24.4%)	118 (31.6%)	291 (26.9%)
**Nulliparous**	337 (47.5%)	213 (57.1%)	550 (50.8%)
**Provider emotionally unavailable**	72 (10.2%)	80 (21.4%)	152 (14.0%)
**Number of support persons present**			
0	0 (0%)	21 (5.6%)	21 (1.9%)
1	636 (89.7%)	333 (89.3%)	969 (89.6%)
2	62 (8.7%)	17 (4.6%)	79 (7.3%)
3+	11 (1.6%)	2 (0.5%)	13 (1.2%)
**Virtual labor support**	25 (3.5%)	47 (12.6%)	72 (6.6%)
**Cesarean section delivery**	191 (26.9%)	110 (29.5%)	301 (27.8%)
**Other childbirth complications**	150 (21.2%)	103 (27.6%)	253 (23.4%)

**Table 2.** Reason support person was missing (participants could select more than one response) (*N* = 346).

Cause	% of cases
Hospital/birth center policy	92.7
No other support people were available to care for other children	6.4
Travel restrictions	5.8
Not allowed due to emergency during the birth (e.g. emergency C-section)	3.7
Birth occurred while the support person was en route	1.8
Other Covid-related reason (e.g. support person ill)	1.6
Other	1.0

During the pregnancy interview, 0 participants said that they would want “no one” to support them in labor and delivery in the absence of restrictions. 98.9% (*N* = 1071) of participants stated that they would want a partner with them at delivery, followed by 31.1% (*N* = 337) who stated that they would want a parent, and 18.9% (*N* = 204) who stated they would want a doula. After delivery, 87.3% of participants reported that their partner attended their birth, while mothers were present at 2.4% (*N* = 29) of births, fathers at 0.2% (*N* = 2) of births, and doulas at 4.5% (*N* = 54). Of the 34% of participants who said that they wished someone else had been in the delivery room with them, mothers were the most frequently missed (45.5% of the subset of 373 participants).

### Regression models

Full model results are provided in Supplementary Tables 1–2. Nulliparity was associated with significantly higher childbirth stress (*B* = 8.4–9.1 across models, all *P* < .001). Education was associated with significantly higher childbirth stress, with a more advanced degree associated with significantly higher reported stress than those without a college degree (*B* = 7.6–8.0 across models, all *P* =< .002). Self-identified race was not associated with perceived birth stress in adjusted models. Cesarean section delivery (*B* = 12–14 across models, all *P* < .001) and other labor and delivery complications (*B* = 17–18 across models, all *P* < 0.001) were also associated with significantly higher childbirth stress.

**Figure 1.**
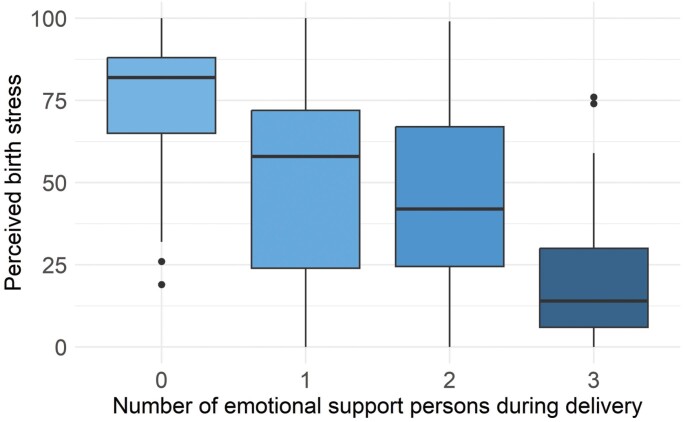
Increased number of emotional support persons in labor was associated with lower perceived childbirth stress, even after adjusting for childbirth complications (*N* = 1082).

We found that five out of six emotional support variables were significantly associated with childbirth stress in the expected direction in both unadjusted and adjusted models (Table 3). Specifically, there was a significant linear relationship between the number of support persons and perceived birth stress (*B* = −20.8, *P* < .001; Fig. 1). The quadratic (*B* = 2.8, *P* = .6) and cubic (*B* = −3.6, *P* = .2) contrasts were not significant, suggesting that a linear trend sufficiently captures the relationship. Individuals who gave birth alone had significantly higher childbirth stress (*B* = 15.7, *P* < .001). Individuals who experienced a mismatch in partner (12.5, *P* = .008) or doula support (*B* = 5.2, *P* = .021) reported significantly higher childbirth stress. Parent mismatch was unrelated to childbirth stress (*B* = 0.85, *P* = .6). Finally, individuals who said that their provider seemed busy, worried, or stressed during delivery had significantly higher childbirth stress (*B* = 16.0, *P* < .001).

**Table 3.** Summary of main effects of emotional support in labor variables predicting perceived childbirth stress across six models (*N* = 1082).

	Unadjusted model			Adjusted model		
	β	95% CI	*P*-value	β	95% CI	*P*-value
Give birth alone	24	12, 36	**<.001**	16	5.4, 27	**.003**
Number of support persons	−33	−47, 20	**<.001**	−21	−33, 8.6	**<.001**
Provider emotional availability	20	16, 25	**<.001**	16	11, 20	**<.001**
Mismatch partner	15	5.1, 26	**.003**	12	3.2, 22	**.008**
Mismatch parent	2.3	−1.4, 6.0	.2	0.85	−2.6, 4.3	.6
Mismatch doula	9.5	4.7, 14	**<.001**	5.2	0.80, 9.6	**.021**

Adjusted models include maternal age, education, race, nulliparity, prenatal depression, cesarean section, and other childbirth complications.

### Sensitivity analyses

Cross-sectional data generally supported the longitudinal trends for the mismatch variables described above. Individuals who said at the postpartum visit that they were missing a desired support person because of the pandemic reported significantly more childbirth stress (Supplementary Table 3, *B* = 7.0, *P* < .001). Individuals who said that they wished their partner (*B* = 14, *P* = 0.004) or doula (*B* = 8.5, *P* = 0.004) had been able to attend the birth and also reported significantly more childbirth stress (Supplementary Table 4). In contrast to the longitudinal analysis, participants who reported that they wished their mother had been able to attend the birth reported significantly higher childbirth stress (*B* = 5.2, *P* = .021).

Having someone attend birth virtually was not significantly associated with reduced childbirth stress (*P* > .37 in both adjusted and unadjusted analyses) and did not significantly attenuate any of the relationships for the other emotional support variables (data not shown).

## Discussion

Humans are characterized by their sociality in all aspects of reproduction, including childbirth. Pelvic changes across human evolutionary history that caused rotational birth could have placed a selective advantage on seeking assistance at delivery [22]. It has been hypothesized that the occiput anterior position of human birth (i.e. most babies are born facing away from the parent, toward the back) makes it difficult for birthing individuals to catch their babies or to remove the umbilical cord from around the baby’s neck [23]. Given the importance of birth attendants for maternal and newborn health, it has been argued that the powerful emotions around labor and birth, such as fear or excitement, could encourage support-seeking behaviors, ultimately enhancing reproductive success [23].

We were therefore interested in understanding whether the evolutionary mismatch resulting from the COVID-19 pandemic—which represented a rapid and somewhat randomly distributed disruption to emotional support persons in labor—would be associated with variation in perceived childbirth stress. We found that five of the six emotional support variables that we tested, including wanting but not having a partner and doula present at delivery, were associated with childbirth stress, even when adjusting for maternal sociodemographic factors and labor and delivery complications. The effect sizes were also substantial, being similar or even greater than those observed for cesarean section and clinical complications. These findings are consistent with the hypothesis that natural selection has shaped a preference for birth attendants to provide in-person emotional support in labor to reduce stress and anxiety [23].

These findings align with and extend previous work. For instance, Preis *et al*. [20] found the absence of an emotional support person during labor was associated with decreased birth satisfaction among US-based individuals during the pandemic. Our study advances this knowledge in several key ways. First, we demonstrate that virtual support failed to mitigate the increased stress associated with missing in-person support, highlighting the irreplaceable nature of physical presence during birth. Second, we identified a previously unreported linear relationship between the number of support people and perceived stress, where each additional support person was associated with lower stress levels. These findings have important implications beyond pandemic contexts, as many hospitals routinely limit support persons to one or two visitors [24]. Given that institutional policies often restrict the number of support persons without clear evidence for these limitations, future research should aim to replicate these findings, with particular attention to potential differences in characteristics among participants who do and do not prefer multiple support persons.

One possible interpretation of our results is that individuals who are more anxious generally are more likely to perceive their births as more stressful and to say that they needed more support in labor, irrespective of the amount of support that was received. While this is possible, the temporal separation between four of our six measures—capturing desired support persons during pregnancy and actual support presence during delivery—helps mitigate concerns about reverse causality. Notably, zero participants expressed a preference during pregnancy for giving birth alone. Therefore, all cases of participants giving birth alone represent an undesired mismatch between preferred and actual support. Similarly, mismatches between desired and actual presence for partners, parents, and doulas were identified by comparing pre-birth preferences to delivery room presence. This prospective study design, combined with the finding that no participants initially desired to give birth alone, strengthens our interpretation that the support person's absence contributed to increased childbirth stress, rather than stress levels influencing retrospective wishes about support.

An additional novel aspect of our study was the evaluation of the perceived attentiveness of the care provider in relation to reported childbirth stress. Fourteen percent of participants perceived their provider as busy or distracted or said that they perceived their provider as limiting their time in the room with them, which could indicate less availability for emotional support. This measure was associated with significantly higher perceived childbirth stress, with a slightly greater magnitude of effect on childbirth stress than cesarean section delivery. While never directly assessed previously, these findings are consistent with the finding that continuous care from a known provider is associated with a more positive birth experience [25]. Such findings have been used to advocate for continuity of care maternity models that are found in cultural contexts such as New Zealand (i.e. in which the same provider meets with the pregnant individual at all stages of prenatal and postpartum care, fostering the development of a trusting relationship), but which are absent from most other cultural contexts, including the USA [26].

A surprising finding in our analysis was that higher education was associated with significantly more childbirth stress in our sample. Additional research is needed to understand whether actual aspects of the birth experience differed according to maternal education or rather whether the perception of those events is what differed, with more educated women feeling relatively less positive about an objectively similar experience.

While our findings support the hypothesis that emotional support during labor has been shaped by natural selection in response to the challenges of human childbirth, an alternative explanation is that the sociality of humans more broadly underlies the desire for emotional support during labor. This alternative view finds some support in studies of three captive and one wild bonobo birth, where researchers observed that females remained in close proximity to the parturient female and demonstrated emotional engagement and supportive behaviors [4, 27]. If similar patterns are observed in more individuals, it could suggest that the evolutionary origins of “midwifery” may predate the specific “obligation” for support that arose from the more difficult, rotational birth process that emerged during hominin evolution.

### Emotional support, stress physiology, and labor

Childbirth is orchestrated by a complex, changing array of interacting hormones—a process shaped by evolution and closely tied to the mental state and emotions of the birthing individual. These physiological effects therefore offer insights into the mechanisms by which emotional support during labor shapes both parental and infant biology and survival, with implications for human evolution. Key hormones involved in this process include oxytocin, epinephrine (adrenaline), and endorphins. Oxytocin causes uterine contractions while also generating calming and analgesic effects during labor and promoting immediate bonding between parent and infant upon delivery [28, 29].

Epinephrine, in contrast, may slow or even reverse labor progress in some cases. This hormone plays a central role in the evolved “fight-or-flight” response and may have enhanced survival during human evolution by slowing or stopping labor in response to perceived danger—especially during early stages [28]. Parallel responses have been observed in experimental studies of other mammals disturbed or stressed during labor, such as mares or cows [30, 31]. Thus, in busy and unfamiliar birth settings laboring individuals may experience elevated epinephrine levels that stall labor progress, even in the absence of direct danger [29]. Conversely, the presence of trusted support people and care providers may promote feelings of calm and safety, thereby facilitating labor progression and reducing the risk of interventions often implemented in cases of “prolonged” labor [22, 32].

Finally, endorphins are endogenous opioids that provide some relief throughout labor, including by potentially altering the birthing person’s state of consciousness to help manage labor-related pain and stress [28, 29]. Endorphins have been linked with feelings of euphoria and reward following delivery and subsequent enhanced parent–infant bonding [28]. The positive effects of endorphins are greatest when individuals feel secure, supported, and are not frightened [29].

Overall, emotional support by attendants who are part of the mother’s community or with whom the mother is familiar can promote labor progression and have a calming physiologic effect [11]. These physiological pathways may help explain the association between social support and perceived birth stress documented in the present study.

### Limitations

Despite the strengths of this manuscript—including uniquely considering the effects of specific support person absence and the influence of the provider on childbirth stress—there are several limitations. First, the survey only asked about the perceived availability of a “provider” generally, and we were, therefore, unable to account for varying levels of support experienced by participants with more than one provider during delivery. Second, we adjusted for both cesarean section and labor and delivery complications, the latter of which is broad and therefore includes complications that vary greatly in terms of clinical significance. We did this because it was the most conservative approach in our analysis. However, future work may choose to evaluate more specific clinical complications in their models. It is also unclear whether or how the order of questioning about perceived stress in the questionnaire could have influenced the results. In addition, due to the use of convenience sampling, these data are not nationally representative. Online surveys may result in biased samples for various reasons, including that they are limited to individuals with internet access, who learn about the survey, who are interested in the topic, and who have the ability to complete it [33]. Thus, white, highly educated individuals are overrepresented in the CARE study sample compared to the US birthing population as a whole [34]. This is consistent with other online surveys conducted during the pandemic, with the shift to online data collection during lockdown resulting in biased samples and non-random attrition in many longitudinal studies [35–37]. The inability to collect data from a nationally representative sample has implications for interpreting study results. While universally beneficial, emotional support in labor is potentially even more important for individuals with elevated risks of adverse birth outcomes, including racialized minorities, individuals with public health insurance, and the uninsured [38]. Specifically, research suggests that doulas in these contexts can enhance health literacy, social support, and quality of care received, in large part by acting as experienced advocates for birthing individuals who are more likely to experience medical mistreatment and encounter inattentive providers [39, 40]. The non-representative nature of the CARE dataset precludes analyses rigorously testing the hypothesized benefits of emotional support during labor in these population sub-groups. More work is therefore needed to assess the impact of emotional support across more representative and diverse samples.

## Conclusions

We found that the absence of any emotional support person during labor—and particularly missing support from a partner, doula, or healthcare provider—was associated with significantly higher perceived childbirth stress. Receiving virtual support did not attenuate these effects. These results align with the hypothesis that human evolution has specifically shaped the need for physical, in-person emotional support during labor. This interpretation is bolstered by previous research suggesting that the evolutionary mismatch of inadequate support during labor increases the risk of cesarean delivery [22, 41].

Given the high rates of cesarean delivery and poor maternal-infant health outcomes in many parts of the world, strategies to improve birth experiences and outcomes are urgently needed. Addressing this evolutionary mismatch by prioritizing adequate emotional support during labor could be a low-risk, low-cost intervention to enhance delivery experiences and outcomes, even outside of public health emergencies like the COVID-19 pandemic. Prioritizing this essential element of the birth process has the potential to yield substantial benefits for mothers, infants, and families.

## Supplementary Material

eoae033_suppl_Supplementary_Material
