# Supplementary material for: Evolutionary mismatch in emotional support during childbirth: Lessons from the COVID-19 pandemic
Source: Evol Med Public Health. 2024 Dec 16;12(1):277–86. doi: 10.1093/emph/eoae033 (PMC11697185; doi:10.1093/emph/eoae033)
Supplement: eoae033_suppl_Supplementary_Material [file eoae033_suppl_supplementary_material.docx]

**Supplementary Information**

**Figure 1.**

**Visual analogue scale question assessing perceived birth stress.**

**
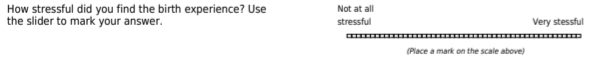
**

**Supplementary Table 1. Adjusted regression analysis predicting birth stress with each emotional support variable (birth alone, number of support persons, provider emotional unavailability) in separate models**

|  | **Birth alone** | | | **Number of support persons** | | | **Provider emotional unavailability** | | |
| --- | --- | --- | --- | --- | --- | --- | --- | --- | --- |
|  | *Beta* | *95% CI* | *p-value* | *Beta* | *95% CI* | *p-value* | *Beta* | *95% CI* | *p-value* |
| **Maternal age** | 0.22 | -0.1, 0.64 | 0.3 | 0.20 | -0.22, 0.61 | 0.4 | 0.24 | -0.16, 0.65 | 0.2 |
| **Education** |  |  |  |  |  |  |  |  |  |
| *No college degree (ref)* | — | — |  | — | — |  | — | — |  |
| *College degree* | 4.2 | -0.59, 9.1 | 0.085 | 4.4 | -0.45, 9.2 | 0.075 | 4.5 | -0.23, 9.2 | 0.062 |
| *Advanced degree* | 7.8 | 3.0, 13 | **<0.001** | 7.6 | 2.9, 12 | **0.002** | 7.7 | 3.0, 12 | **0.001** |
| **Race** |  |  |  |  |  |  |  |  |  |
| *White (ref)* | — | — |  | — | — |  | — | — |  |
| *Hispanic* | 6.9 | -0.11, 14 | 0.054 | 6.8 | -0.26, 14 | 0.059 | 6.2 | -0.68, 13 | 0.077 |
| *Black/African American* | 12 | -2.3, 26 | 0.10 | 12 | -2.5, 26 | 0.11 | 12 | -2.2, 26 | 0.10 |
| *Asian* | -4.7 | -14, 4.5 | 0.3 | -5.2 | -14, 4.1 | 0.3 | -4.6 | -14, 4.5 | 0.3 |
| *American Indian/Alaska Native* | 15 | -4.0, 34 | 0.12 | 15 | -4.5, 34 | 0.13 | 11 | -8.1, 30 | 0.3 |
| *Other* | -5.3 | -17, 6.8 | 0.4 | -4.7 | -17, 7.3 | 0.4 | -4.0 | -16, 7.9 | 0.5 |
| **Nulliparous** | 9.1 | 5.9, 12 | **<0.001** | 8.7 | 5.4, 12 | **<0.001** | 8.5 | 5.3, 12 | **<0.001** |
| **Prenatal depression** | 9.2 | 5.7, 13 | **<0.001** | 9.1 | 5.6, 13 | **<0.001** | 7.6 | 4.1, 11 | **<0.001** |
| **Childbirth complications** | 18 | 14, 21 | **<0.001** | 18 | 14, 22 | **<0.001** | 17 | 13, 20 | **<0.001** |
| **Caesarean delivery** | 13 | 9.1, 16 | **<0.001** | 12 | 8.7, 16 | **<0.001** | 14 | 10, 17 | **<0.001** |
| **Give birth alone** | 16 | 5.4, 27 | **0.003** |  |  |  |  |  |  |
| **Number of support persons** |  |  |  | -21 | -33, 8.6 | <0.001 |  |  |  |
| **Provider emotional availability** |  |  |  |  |  |  | 16 | 11, 20 | **<0.001** |
| **Model Adj R^2^** |  |  | 0.21 |  |  | 0.21 |  |  | 0.23 |

**Supplementary Table 2. Adjusted regression analysis predicting birth stress with each emotional support variable (mismatch partner, mismatch parent, mismatch doula) in separate models**

|  | **Mismatch Partner** | | | **Mismatch Parent** | | | **Mismatch Doula** | | |
| --- | --- | --- | --- | --- | --- | --- | --- | --- | --- |
|  | *Beta* | *95% CI* | *p-value* | *Beta* | *95% CI* | *p-value* | *Beta* | *95% CI* | *p-value* |
| **Maternal age** | 0.21 | -0.20, 0.63 | 0.3 | 0.18 | -0.24, 0.60 | 0.4 | 0.23 | -0.19, 0.65 | 0.3 |
| **Education** |  |  |  |  |  |  |  |  |  |
| *No college degree (ref)* | — | — |  | — | — |  | — | — |  |
| *College degree* | 4.4 | -0.45, 9.2 | 0.076 | 4.1 | -0.77, 8.9 | 0.10 | 4.3 | -0.52, 9.2 | 0.080 |
| *Advanced degree* | 8.0 | 3.2, 13 | **0.001** | 7.8 | 3.0, 13 | **0.001** | 8.0 | 3.3, 13 | **0.001** |
| **Race** |  |  |  |  |  |  |  |  |  |
| *White (ref)* | — | — |  | — | — |  | — | — |  |
| *Hispanic* | 6.8 | -0.26, 14 | 0.059 | 6.5 | -0.53, 14 | 0.070 | 6.8 | -0.24, 14 | 0.058 |
| *Black/African American* | 12 | -2.0, 26 | 0.091 | 13 | -1.0, 27 | 0.069 | 13 | -1.7, 27 | 0.085 |
| *Asian* | 4.8 | -14, 4.5 | 0.3 | -5.5 | -15, 3.8 | 0.2 | -5.1 | -14, 4.2 | 0.3 |
| *American Indian/Alaska Native* | 15 | -4.0, 34 | 0.12 | 13 | -6.6, 32 | 0.2 | 15 | -4.6, 34 | 0.14 |
| *Other* | -5.3 | -17, 6.8 | 0.4 | -5.4 | -17, 6.7 | 0.4 | -5.8 | -18, 6.3 | 0.4 |
| **Nulliparous** | 9.1 | 5.9, 12 | **<0.001** | 8.4 | 5.1, 12 | **<0.001** | 8.7 | 5.4, 12 | **<0.001** |
| **Prenatal depression** | 9.2 | 5.7, 13 | **<0.001** | 9.0 | 5.5, 13 | **<0.001** | 9.2 | 5.6, 13 | **<0.001** |
| **Childbirth complications** | 18 | 14, 22 | **<0.001** | 18 | 14, 22 | **<0.001** | 18 | 14, 22 | **<0.001** |
| **Caesarean delivery** | 13 | 9.3, 16 | **<0.001** | 13 | 9.6, 17 | **<0.001** | 13 | 9.9, 17 | **<0.001** |
| **Mismatch partner** | 12 | 3.2, 22 | **0.008** |  |  |  |  |  |  |
| **Mismatch parent** |  |  |  | 5.2 | 0.80, 9.6 | **0.021** |  |  |  |
| **Mismatch doula** |  |  |  |  |  |  | 0.85 | -2.6, 4.3 | 0.6 |
| **Model Adj R^2^** |  |  | 0.20 |  |  | 0.20 |  |  | 0.20 |

**Sensitivity analyses**

**Supplementary Table 3. Relationship between saying that you were missing support a person in labor (measured at postpartum survey) and childbirth stress.**

| **Characteristic** | **Beta** | **95% CI** | **p-value** |
| --- | --- | --- | --- |
| Maternal age | 0.25 | -0.17, 0.66 | 0.2 |
| Education (ref: no college degree) |  |  |  |
| *College degree* | 4.8 | -0.03, 9.6 | 0.052 |
| *Advanced degree* | 8.6 | 3.8, 13 | **<0.001** |
| Race (ref: white) |  |  |  |
| *Hispanic* | 6.5 | -0.51, 13 | 0.069 |
| *Black/African American* | 12 | -2.3, 26 | 0.10 |
| *Asian* | 12 | -2.3, 26 | 0.10 |
| *AI/AN* | 13 | -6.3, 32 | 0.2 |
| *Other* | -5.5 | -17, 6.5 | 0.4 |
| *Nulliparous* | 8.3 | 5.1, 11 | **<0.001** |
| Prenatal depression | 8.6 | 5.1, 12 | **<0.001** |
| Childbirth complications | 18 | 14, 21 | **<0.001** |
| Caesarean delivery | 13 | 9.8, 17 | **<0.001** |
| ***Missing support person*** | 6.7 | 3.4, 9.9 | **<0.001** |

**Supplementary Table 4. Relationship between saying that you were missing a specific support person in labor (measured at postpartum visit) and childbirth stress.**

|  | Missing partner | | | Missing mother | | | Missing doula | | |
| --- | --- | --- | --- | --- | --- | --- | --- | --- | --- |
|  | **Beta** | **95% CI** | **p-value** | **Beta** | **95% CI** | **p-value** | **Beta** | **95% CI** | **p-value** |
| Maternal age | 0.22 | -0.20, 0.63 | 0.3 | 0.28 | -0.14, 0.70 | 0.2 | 0.17 | -0.24, 0.59 | 0.4 |
| Education (ref: no college) |  |  |  |  |  |  |  |  |  |
| *College degree* | 4.6 | -0.25, 9.4 | 0.063 | 4.7 | -0.14, 9.5 | 0.057 | 4.3 | -0.50, 9.1 | 0.079 |
| *Advanced degree* | 8.1 | 3.3, 13 | **<0.001** | 8.4 | 3.7, 13 | **<0.001** | 7.8 | 3.1, 13 | **0.001** |
| Race (ref: white) |  |  |  |  |  |  |  |  |  |
| *Hispanic* | 6.8 | -0.25, 14 | 0.059 | 6.5 | -0.53, 14 | 0.070 | 6.3 | -0.72, 13 | 0.079 |
| *Black/African American* | 12 | -2.0, 26 | 0.092 | 12 | -2.3, 26 | 0.10 | 14 | -0.53, 28 | 0.059 |
| *Asian* | -4.8 | -14, 4.5 | 0.3 | -5.1 | -14, 4.2 | 0.3 | -5.9 | -15, 3.4 | 0.2 |
| *AI/AN* | 13 | -5.9, 33 | 0.2 | 14 | -5.1, 33 | 0.2 | 13 | -6.0, 32 | 0.2 |
| *Other* | -5.2 | -17, 6.8 | 0.4 | -6.3 | -18, 5.7 | 0.3 | -4.7 | -17, 7.3 | 0.4 |
| *Nulliparity* | 9.1 | 5.9, 12 | **<0.001** | 8.4 | 5.2, 12 | **<0.001** | 8.3 | 5.1, 12 | **<0.001** |
| Prenatal depression | 9.1 | 5.6, 13 | **<0.001** | 9.0 | 5.5, 13 | **<0.001** | 8.9 | 5.4, 12 | **<0.001** |
| Childbirth complications | 18 | 14, 22 | **<0.001** | 18 | 14, 22 | **<0.001** | 18 | 14, 21 | **<0.001** |
| Caesarean delivery | 13 | 9.3, 16 | **<0.001** | 13 | 10, 17 | **<0.001** | 13 | 9.9, 17 | **<0.001** |
| Missing partner | 14 | 4.5, 23 | **0.004** |  |  |  |  |  |  |
| Missing mother |  |  |  | 5.7 | 1.9, 9.5 | **0.003** |  |  |  |
| Missing doula |  |  |  |  |  |  | 8.5 | 2.8, 14 | **0.004** |
